# Sodium zirconium cyclosilicate post hospital discharge to prevent hyperkalaemia: phase 4, randomized CONTINUITY trial

**DOI:** 10.1093/ckj/sfag158

**Published:** 2026-05-19

**Authors:** James O Burton, María J Izquierdo, Cecilia Linde, Nicolás R Robles, Manish M Sood, Alaster M Allum, James M Eudicone, Magnus Dahl, Alpesh N Amin

**Affiliations:** Division of Cardiovascular Sciences, University of Leicester and University Hospitals of Leicester, Leicester, UK; Head of Nephrology Department, University Hospital of Burgos, Burgos, Spain; Department of Cardiology, Karolinska Institutet, Stockholm, Sweden; Universidad de Extremadura, Head Nephrology Department and Hypertension Unit, Hospital Universitario de Badajoz, Badajoz, Spain; Ottawa Hospital Research Institute, The Ottawa Hospital, Ottawa, Canada; BioPharmaceuticals Medical, AstraZeneca, Cambridge, UK; BioPharmaceuticals Medical Evidence, AstraZeneca, Wilmington, DE, USA; BioPharmaceuticals Medical, AstraZeneca, Gothenburg, Sweden; Department of Medicine, University of California Irvine, Irvine, CA, USA

**Keywords:** CKD, hospitalization, hyperkalaemia, potassium binder, renin-angiotensin-aldosterone system inhibitor

## Abstract

**Background and hypothesis:**

Hyperkalaemia is common and potentially life-threatening in individuals with chronic kidney disease (CKD). While sodium zirconium cyclosilicate (SZC) is used in the hospital setting for hyperkalaemia management, few patients are discharged with a continuing prescription, leaving them at risk of recurrent hyperkalaemia and rehospitalization. The CONTINUITY study aimed to establish the efficacy of continuing post-hospital discharge treatment with SZC versus standard of care (SOC) in maintaining normokalaemia and reducing all-cause and hyperkalaemia-related hospital admissions and emergency department visits in patients with CKD and hyperkalaemia.

**Methods:**

This phase 4, randomized, controlled, open-label, parallel-group (multicentre study 28 sites; six European countries; 24 March 2022–10 December 2024), included participants aged ≥18 years admitted to hospital with diagnosed CKD (any stage) and hyperkalaemia [serum potassium (sK^+^) >5.0 and ≤6.5 mmol/l] without ongoing K^+^ binder treatment. Participants received SZC for 2–21 days to normalize sK^+^. Those with normokalaemia (3.5–5.0 mmol/l) at discharge were randomized to SZC or SOC. The primary objective was occurrence of normokalaemia at 180 days post-discharge [those achieving normokalaemia (responders) vs those who did not (non-responders)].

**Results:**

The difference between arms for normokalaemia responders was not statistically significant: 30.9% (SZC) and 36.2% (SOC) (odds ratio 0.81; 95% CI 0.39–1.66; *P* = .558). High and imbalanced levels of missing sK^+^ measurements at 180 days [*n* = 35 (51.5%) in the SZC arm and *n* = 25 (36.2%) in the SOC arm] led to substantially lower levels of normokalaemia response than the original assumptions in the sample size calculations used to power the study.

**Conclusion:**

The study did not meet its primary endpoint. Study design limitations may have masked true treatment effects. Post hoc analyses revealed positive trends in SZC use and potential for reducing rehospitalization and enabling renin-angiotensin-aldosterone system inhibitor therapy continuation at optimal doses.

## Graphical Abstract

**Graphical Abstract:**
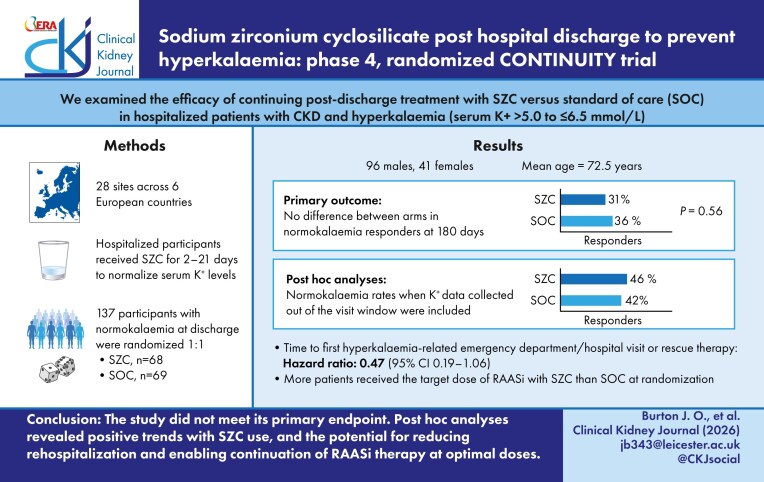

KEY LEARNING POINTS **What was known:**

- Hyperkalaemia is a common and potentially life-threatening situation in individuals with chronic kidney disease (CKD).
- While sodium zirconium cyclosilicate (SZC) is used in the hospital setting for hyperkalaemia management, few patients are discharged with a continuing prescription, leaving them at risk of recurrent hyperkalaemia and rehospitalization.
- Whether SZC prevents recurrence of hyperkalaemia, and reduces hospitalization and rehospitalization rates, is unclear.

**This study adds:**

- This multinational trial in 137 adults with CKD showed no difference between SZC and standard of care regarding participants with normokalaemia 180 days post hospital discharge.
- Post hoc analysis revealed positive trends in SZC use.

**Potential impact:**

- While the study did not meet its primary endpoint, post hoc analysis indicated potential benefits of continued SZC in the outpatient setting with regard to all-cause hospitalized patients with CKD and hyperkalaemia.

Hyperkalaemia, defined as a serum potassium (sK^+^) level >5.0–5.5 mmol/l [1, 2], is an increasingly prevalent and potentially life-threatening situation, frequently seen as a medical emergency [1, 2]. It is common among individuals with chronic kidney disease (CKD), with an estimated event rate of 6.9 out of 100 person-years for those with an estimated glomerular filtration rate (eGFR) <30 ml/min/1.73 m^2^ [3], and is a common cause of, or occurs concurrently during, hospitalization [4–6].

Renin-angiotensin-aldosterone system inhibitors (RAASi) are evidence-based medications for cardiorenal protection [7]; however, they are an established cause of elevations in sK^+^ [8]. They are therefore frequently down-titrated or discontinued when hyperkalaemia occurs [9, 10], which is contraindicative to international guideline recommendations and increases the risk of major adverse cardiac events and death [11]. Many individuals are not reinstated on appropriate RAASi doses, representing a missed opportunity for disease prevention [12–14].

Sodium zirconium cyclosilicate (SZC) is a novel, orally administered K^+^ binder that rapidly lowers and maintains sK^+^ levels within a normal range (3.5–5.0 mmol/l) [15–18]. SZC is frequently used in the hospital setting to reduce acute rises in sK^+^. However, few patients are discharged with a continuing prescription [19], which may leave them vulnerable to recurrent hyperkalaemia episodes and rehospitalization. Recent evidence suggests that the use of SZC in the outpatient setting enables continuation and optimization of RAASi therapy [18, 20, 21]. However, whether SZC prevents recurrence of hyperkalaemia, and reduces hospitalization and rehospitalization rates, remains unclear.

Here, we present the results from the randomized, phase 4, CONTINUITY study (ClinicalTrials.gov: NCT05347693) [22], which evaluated the efficacy of continuing post-discharge treatment with SZC versus standard of care (SOC) in maintaining normokalaemia and reducing hyperkalaemia-related hospital admissions and emergency department (ED) visits in all-cause hospitalized patients with CKD and hyperkalaemia.

## MATERIALS AND METHODS

### Study design and ethics

The CONTINUITY study rationale and design have been published previously [23]; the study protocol and statistical analysis plan are available in the Supplementary data. Briefly, CONTINUITY was a phase 4, randomized, controlled, open-label, parallel-group, multicentre study in participants with CKD who were hospitalized with hyperkalaemia. All participants underwent an ‘active treatment for hyperkalaemia’, meaning they received SZC for between 2 and 21 days to normalize sK^+^ levels (10 g three times daily for up to 72 hr and, once sK^+^ was between 3.5 and 5.0 mmol/l, 5 g once daily adjusted per local label). Those with normokalaemia (3.5–5.0 mmol/l) at hospital discharge were randomized to receive SZC (dose per local label) or SOC and constituted the study population. The definition of SOC was at the discretion of the treating physician and per local practice. The study was conducted in 28 sites across six European countries (Belgium, France, Italy, Netherlands, Spain, and the United Kingdom) between 24 March 2022 and 10 December 2024.

The study was approved by all relevant ethics committees [Belgium: Ethical committee research UZ/KU Leuven (S66639); France: Comité de protection des personnes Sud-Est V (21-ASTR-01); Italy: Comitato Etico Territoriale Lombardia 6 (#2606); Netherlands: Netherlands Medical Ethics Review Committee (METc) 2021/700 (M22.306049); Spain: CEIM Provincial De Sevilla, ORG–100032546 (20/2021); UK: London – Riverside Research Ethics Committee (22/LO/0020)]. The study also adhered to consensus ethical principles derived from international guidelines, including the Declaration of Helsinki, the Council for International Organizations of Medical Sciences International Ethical Guidelines, the International Council for Harmonisation Good Clinical Practice Guidelines, and applicable laws and regulations. All participants were required to sign an informed consent form. Patients or the public were not involved in the design, conduct, reporting, or dissemination plans of this research.

### Inclusion and exclusion criteria

The inclusion and exclusion criteria have been published previously [23]. To allow for the inclusion of a wider and more applicable patient population, key protocol updates were made, including a broader eGFR inclusion criterium (Supplementary Methods and Supplementary Fig. S1). Briefly, eligible participants were adults aged ≥18 years who were admitted to hospital with diagnosed CKD (any stage) and hyperkalaemia (sK^+^ >5.0 and ≤6.5 mmol/l) in the absence of ongoing K^+^ binder treatment. CKD was defined as eGFR <90 ml/min/1.73 m^2^ at, or within 3 months of, study screening, based on the Chronic Kidney Disease Epidemiology Collaboration equation (race and ethnicity were not included in the equation). As per the updated protocol, participants were eligible if they had normokalaemia, as long as they were receiving treatment for the current episode of hyperkalaemia. This treatment could be a K^+^ binder such as SZC or patiromer, but it could also be other treatments for hyperkalaemia, such as diuretics, insulin, and sodium bicarbonate.

### Endpoints

The study objectives and endpoints have been published previously [23]. The primary study objective was to evaluate the efficacy of continuing SZC treatment as part of the discharge medications versus SOC in maintaining normokalaemia (sK^+^ 3.5–5.0 mmol/l) at 180 days post-discharge (responder achieving normokalaemia vs non-responder who did not). Normokalaemia non-response was defined as any of the following: use of rescue therapy for hyperkalaemia; down-titration (including discontinuation) of RAASi treatment [i.e. angiotensin-converting enzyme inhibitors (ACEis), angiotensin receptor blockers (ARBs), and mineralocorticoid receptor antagonists (MRAs)]; all-cause death before 180 days post-discharge; participants lost to follow-up, who withdrew consent, or stopped SZC treatment due to adverse events (AEs); participants whose final sK^+^ reading was outside the 1-week visit window; or participants withdrawn due to site decision.

The main study secondary objective was the effectiveness of continuing SZC as part of the discharge medications versus SOC in reducing the incidence of the composite outcome of all-cause hospital admissions, ED visits with hyperkalaemia as a contributing factor, all-cause death, or use of rescue therapy for hyperkalaemia at any time up to 180 days post-discharge. Protocol updates to the secondary objective are summarized in the Supplementary Methods and Supplementary Fig. S1.

### Statistical analysis

Details of the statistical methodology have been published previously [23]. The sample size calculation for this study used the updated main secondary endpoint, rather than the primary study endpoint as the number of participants required was smaller, and was based on the log-rank test for equality of survival curves. Using prior data, it was assumed that 1% of participants in the SZC group and 7% of participants in the SOC group would experience an event from the composite outcome. Assuming a 20% initial screening failure rate to the in-hospital phase, a 15% screening failure rate from the in-hospital phase to the discharge visit, and a 20% drop-out post-randomization, a total of 632 participants were needed to be screened and 430 participants randomized (215 per arm) to achieve 344 evaluable participants (172 per arm). For assessment of the primary study endpoint, based on these screening failure rates, and assuming the proportions of participants with normokalaemia at 180 days were 0.88 (SZC) and 0.59 (SOC), 132 participants needed to be screened.

For the primary endpoint, occurrence (responder or non-responder) of normokalaemia at 180 days post-discharge was analysed using a logistic regression model, with response as the dependent variable and randomized treatment group as the independent variable. For participants who discontinued treatment with an sK^+^ measurement at 180 days post-discharge, this value was used irrespective of treatment discontinuation.

Sensitivity analyses evaluated the impact of considering non-response versus only evaluating completers.

Analyses for the primary, secondary, and exploratory endpoints used the full analysis set (all randomized participants), while safety analyses used the randomized safety set (all randomized participants who received at least one dose of SZC post-discharge in the SZC arm, and all randomized participants in the SOC arm) [23]. The odds ratio (OR), along with the two-sided 95% confidence intervals (CIs) and two–sided *P*-value (where *P* < .05 indicated statistical significance), were reported.

To control for type I error, a hierarchical testing procedure was followed when formally analysing the primary and secondary endpoints. This procedure followed a stepwise algorithm where each endpoint was only formally tested if the preceding null hypothesis was rejected (*P* < .05). If the preceding null hypothesis was not rejected, then the evaluation of the endpoint was reduced to that of the exploratory endpoint.

Safety and tolerability were evaluated in terms of AEs, serious AEs (SAEs), AEs leading to treatment discontinuation, clinical laboratory data, vital signs, and electrocardiogram data. AEs were classified using the Medical Dictionary for Regulatory Activities system organ class and preferred term. The extent of missing data was reported [23].

### Post hoc analysis

Post hoc analyses included analysis of sK^+^ data from participants with sK^+^ readings outside the specified 1-week visit window at 180 days post-discharge; normokalaemia responders/non–responders at 90 days; use and dose of RAASi therapy (including at randomization, and at 90 and 180 days post-discharge); time to first occurrence of hyperkalaemia-related events including hospital admission with hyperkalaemia as a contributing factor, ED visit with hyperkalaemia, or use of rescue therapy for hyperkalaemia; and proportion of participants with hyperkalaemia (including at randomization, and at 90 and 180 days post-discharge).

## RESULTS

### Participant disposition and demographic characteristics

Participant disposition is summarized in Supplementary Fig. S2 and baseline characteristics and demographics of the randomized participants at screening are shown in Table 1 and Supplementary Table S1. Of the 186 participants enrolled and screened from 28 sites, 137 were randomized 1:1 to receive treatment with SZC (*n* = 68) or SOC (*n* = 69).

**Table 1:** Baseline demographics and patient characteristics (full analysis set).

Baseline characteristic	SZC (*n* = 68)	SOC (*n* = 69)	Total (*N* = 137)
Age (years), mean (SD)	72.8 (10.3)	72.2 (10.9)	72.5 (10.6)
≥65 years	54 (79.4)	56 (81.2)	110 (80.3)
Female	25 (36.8)	16 (23.2)	41 (29.9)
Race			
White	64 (94.1)	65 (94.2)	129 (94.2)
Asian	0	1 (1.4)	1 (0.7)
Not reported	4 (5.9)	3 (4.3)	7 (5.1)
Ethnic group			
Hispanic or Latino	21 (30.9)	10 (14.5)	31 (22.6)
Not Hispanic or Latino	43 (63.2)	57 (82.6)	100 (73.0)
Country of origin			
Belgium	0	2 (2.9)	2 (1.5)
France	4 (5.9)	2 (2.9)	6 (4.4)
Italy	6 (8.8)	3 (4.3)	9 (6.6)
Netherlands	1 (1.5)	0	1 (0.7)
Spain	57 (83.8)	60 (87.0)	117 (85.4)
United Kingdom	0	2 (2.9)	2 (1.5)
SK^+a^			
≤5.0 mmol/l	14 (20.6)	14 (20.9)	28 (20.7)
>5.0 to ≤5.5 mmol/l (mild)	33 (48.5)	32 (47.8)	65 (48.1)
>5.5 to ≤6.0 mmol/l (moderate)	16 (23.5)	16 (23.9)	32 (23.7)
>6.0 to ≤6.5 mmol/l (severe)	5 (7.4)	5 (7.4)	10 (7.4)
CKD stage^b^			
Stage 1	0	0	0
Stage 2	2 (2.9)	1 (1.5)	3 (2.2)
Stage 3	18 (26.5)	22 (32.3)	40 (29.4)
Stage 4	34 (50.0)	29 (42.6)	63 (46.3)
Stage 5	14 (20.6)	12 (17.6)	26 (19.1)
Unknown	0	4 (5.8)	4 (2.9)
Use of ACEi/ARB/ARNi/MRA	55 (80.9)	49 (71.0)	104 (75.9)

Data are presented as *n* (%) unless otherwise stated.

a Percentages calculated based on the number of participants with non-missing sK^+^ values at baseline for each treatment group. Data missing for two participants in the SOC group. Most baseline sK^+^ values were assessed at the screening visit.

b Additional characteristics were recorded from the randomized safety set, which included 68 participants each in the SZC and SOC groups.

ACEi, angiotensin-converting enzyme inhibitor; ARB, angiotensin receptor blocker; ARNi, angiotensin receptor-neprilysin inhibitor; CKD, chronic kidney disease; eGFR, estimated glomerular filtration rate; MRA, mineralocorticoid receptor antagonist; sK^+^, serum potassium; SOC, standard of care; SZC, sodium zirconium cyclosilicate.

Overall, mean age was 72.5 years, with 80.3% of participants aged ≥65 years or older and 29.9% being female. In the SZC group versus the SOC group, there were slightly more females (SZC: 36.8%; SOC: 23.2%). Most participants originated from Spain (85.4%), 94.2% were White, and 22.6% were Hispanic or Latino (SZC: 30.9%; SOC: 14.5%).

A high proportion of patients had CKD stage 4–5 (65.4%). There were ∼10% more patients with CKD stage 4/5 in the SZC versus the SOC group [stage 4: 34 (50.0%) vs 29 (42.6%); stage 5: 14 (20.6%) vs 12 (17.6%)]. In the SZC versus the SOC group, diabetes was present in 64.7% versus 57.4% of participants; the presence of heart failure was comparable between the two groups (22.1% vs 20.6%) (Table 1 and Supplementary Table S1). At baseline, 48.1% of participants had mild hyperkalaemia (sK^+^ >5.0 to ≤5.5 mmol/l) and 31.1% had moderate/severe hyperkalaemia (sK^+^ >5.5 to ≤6.5 mmol/l).

The overall mean [standard deviation (SD)] study treatment compliance was 96.2% (37.3) during the in-patient phase and 107.8% (66.0) during the post-discharge phase. Mean (SD) treatment compliance to SZC was 110.1% (68.1) during the post-discharge phase.

### Primary endpoint

**Figure 1:**
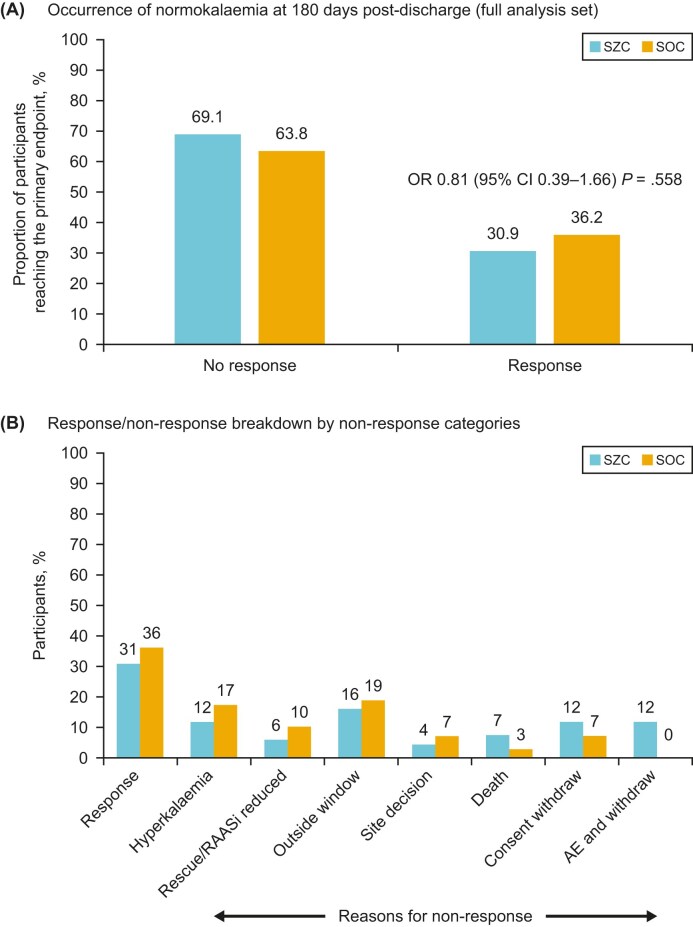
Occurrence (response/no response) of (A) normokalaemia at 180 days post-discharge (full analysis set) and (B) normokalaemia response/non-response breakdown by non-response categories. Use of rescue therapy, down-titration, or discontinuation of RAASi, death, or lost to follow-up, and participants who withdrew, site decision to withdraw participants, or those with serum potassium reading outside the 1-week visit window were also counted as non-response. AE, adverse event; OR, odds ratio; RAASi, renin-angiotensin-aldosterone system inhibitor; SOC, standard of care; SZC, sodium zirconium cyclosilicate.

No statistically significant difference was found between the SZC versus SOC arms in terms of responders (i.e. patients with normokalaemia at 180 days post-discharge): 30.9% versus 36.2%, respectively; OR 0.81; 95% CI 0.39–1.66; *P* = .558 (Fig. 1A). In the primary analysis, there were high and imbalanced levels of missing sK^+^ measurements at 180 days [*n* = 35 (51.5%) in the SZC arm and *n* = 25 (36.2%) in the SOC arm].

Sensitivity analyses were consistent and showed no evidence of a statistically significant difference between SZC and SOC arms (data not shown).

Figure 1B demonstrates normokalaemia non-responders at 180 days with reasons for non-response. The most common reason for participants being non-responders was having their end-of-treatment (180 days post-discharge) sK^+^ reading outside of the 1-week visit window (SZC, 16.2%; SOC, 18.8%).

The null hypothesis was not rejected for the primary endpoint, so the hierarchical testing procedure was not followed for the evaluation of subsequent endpoints; therefore, all further endpoints were considered exploratory.

### Secondary endpoint

**Figure 2:**
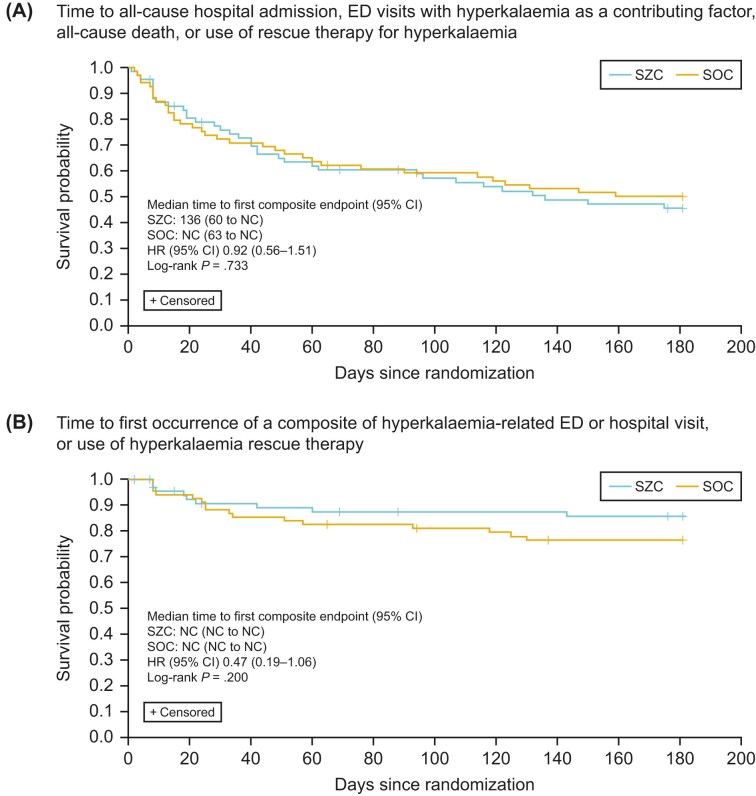
Kaplan-Meier analysis of time to first occurrence of (A) all-cause hospital admission, ED visits with hyperkalaemia as a contributing factor, all-cause death, or use of rescue therapy for hyperkalaemia, and (B) hospital admission with hyperkalaemia as a contributing factor, ED visits with hyperkalaemia, or use of rescue therapy for hyperkalaemia (full analysis set). ED, emergency department; HR, hazard ratio; NC, not calculated; SOC, standard of care; SZC, sodium zirconium cyclosilicate.

No difference was found between the SZC versus SOC arms in the time to first occurrence of all-cause hospital admission, ED visits with hyperkalaemia as a contributing factor, all-cause death, or use of rescue therapy for hyperkalaemia: 51.5% versus 49.3%, respectively; hazard ratio (HR) 0.92; 95% CI 0.56–1.51; *P* = .733 (Fig. 2A).

### Safety

The proportion of participants with an AE was comparable between the SZC and SOC arms (73.5% and 76.5%, respectively; Table 2). Hyperkalaemia was present in 19.1% of participants in the SZC arm and 27.9% in the SOC arm. Numerically, more participants in the SZC arm experienced an SAE compared with those in the SOC arm (42.6% vs 30.9%), which was mainly driven by gastrointestinal disorders (5.9% vs 1.5%—all preferred terms occurred in *n* = 1 participants).

**Table 2:** Summary of AEs (safety analysis set).

	*N* (%)	
Event	SZC (*n* = 68)	SOC (*n* = 68)
Any AE	50 (73.5)	52 (76.5)
Blood and lymphatic system disorders	6 (8.8)	9 (13.2)
Anaemia	4 (5.9)	9 (13.2)
Cardiac disorders	9 (13.2)	10 (14.7)
Cardiac failure	6 (8.8)	4 (5.9)
Gastrointestinal disorders	13 (19.1)	7 (10.3)
Diarrhoea	4 (5.9)	1 (1.5)
Infections and infestations	17 (25.0)	16 (23.5)
Urinary tract infection	3 (4.4)	7 (10.3)
Metabolism and nutritional disorders	18 (26.5)	25 (36.8)
Hyperkalaemia	13 (19.1)	19 (27.9)
Metabolic acidosis	3 (4.4)	4 (5.9)
Renal and urinary disorders	13 (19.1)	13 (19.1)
Renal impairment	5 (7.4)	4 (5.9)
Chronic kidney disease	3 (4.4)	4 (5.9)
Acute kidney injury	5 (7.4)	1 (1.5)
Any AE assessed by investigator as possibly related to treatment	9 (13.2)	0
Any AE with an outcome of death	6 (8.8)	2 (2.9)
Any AE leading to treatment discontinuation	13 (19.1)	0
Any AE leading to dose interruption	6 (8.8)	0
Any AE leading to dose reduction	1 (1.5)	0
Any SAE (including events with an outcome of death)	29 (42.6)	21 (30.9)
Cardiac disorders	5 (7.4)	5 (7.4)
Gastrointestinal disorders	4 (5.9)	1 (1.5)
Infections and infestations	9 (13.2)	10 (14.7)
Renal and urinary disorders	4 (5.9)	3 (4.4)
Any SAE leading to treatment discontinuation	7 (10.3)	0

AE, adverse event; SAE, serious adverse event; SOC, standard of care; SZC, sodium zirconium cyclosilicate.

There were numerically more deaths in the SZC arm [*n* = 6 (8.8%); septic shock, myasthenia gravis crisis, cardiac arrest, cardiac failure, acute pulmonary oedema, and acute kidney injury] than the SOC arm [*n* = 2 (2.9%); COVID-19 and cardiac tamponade]. All deaths were adjudicated by the safety team and found to be unrelated to the study medication.

In the SZC arm, 13 patients had AEs and seven had SAEs that led to treatment discontinuation. No AEs or SAEs led to treatment discontinuation in the SOC arm.

### Post hoc analysis

#### Analysis including participants with sK^+^ readings outside the 1-week visit window at 180 days post-discharge

When sK^+^ data collected outside of the 1-week visit window at 180 days were included (rather than treated as non-responders), response (occurrence of normokalaemia) was achieved in 45.6% of participants in the SZC arm and 42.0% of participants in the SOC arm, with an OR (95% CI) of 1.19 (0.60–2.37).

#### Responders and non-responders at 90 days post-discharge

At 90 days, normokalaemia response rates were 61.8% in the SZC arm and 53.6% in the SOC arm. At 90 days, 15 (22.1%) participants in the SZC arm and six (8.7%) in the SOC arm had missing sK^+^ measurements. The main reason for non-response was sK^+^ outside the 3.5–5.0-mmol/l range (SZC, 16.2%; SOC, 37.7%); rates of the other non–response categories were lower than at 180 days (data not shown).

In the per-protocol population at 90 days, post hoc analysis when sK^+^ data collected outside of the 1-week visit window at 180 days were included showed a treatment effect in favour of SZC (*n* = 53; responders, 60.4%) versus SOC (*n* = 61; responders, 42.6%), with an OR (95% CI) of 2.14 (1.00–4.58; nominal *P* = .051).

#### RAASi use and dose

**Figure 3:**
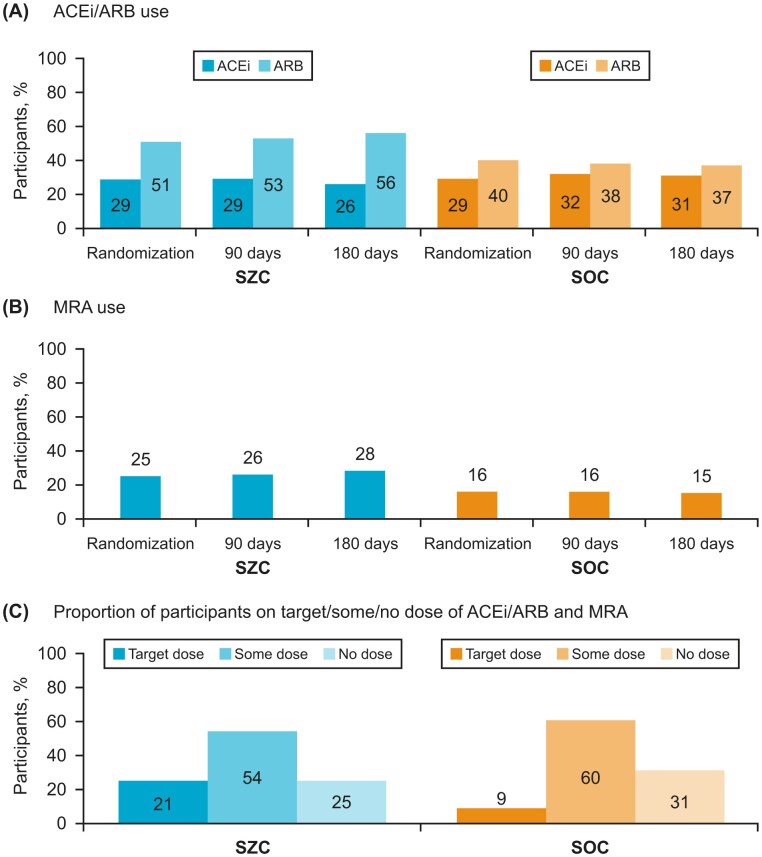
(A) ACEi/ARB and (B) MRA use between randomization and 180 days post-discharge, and (C) proportion of participants on doses of RAASi therapy at randomization (full analysis set) ACEi, angiotensin-converting enzyme inhibitor; ARB, angiotensin receptor blocker; MRA, mineralocorticoid receptor antagonist; RAASi, renin-angiotensin-aldosterone system inhibitor; SOC, standard of care; SZC, sodium zirconium cyclosilicate.

At randomization, a lower proportion of participants were receiving ARB treatment in the SOC arm (ARB 40%) compared with the SZC arm (ARB 51%) (Fig. 3A). ACEi/ARB use increased slightly in the SZC group at 90 and 180 days but decreased slightly in the SOC group. A similar trend was observed with MRA use, with approximately 36% fewer participants in the SOC arm (16%) compared with the SZC arm (25%) receiving MRAs at randomization (Fig. 3B).

Approximately twice as many participants in the SZC arm (21%) compared with the SOC arm (9%) were at target dose for at least one ACEi/ARB/MRA at randomization (Fig. 3C). The proportions of patients on target doses of ACEis, ARBs, or MRAs at 90 days and 180 days are shown in Supplementary Table S2.

#### Time to occurrence of first composite hyperkalaemia-related event

The median time to the first occurrence of a composite of hyperkalaemia-related ED or hospital visit, or use of hyperkalaemia rescue therapy, did not differ between the two study arms (HR 0.47; 95% CI 0.19–1.06; nominal *P* = .200) (Fig. 2B).

#### Proportion of participants with hyperkalaemia at randomization, and at 90 days and 180 days post-discharge

Numerically more participants in the SOC arm experienced hyperkalaemia at days 90 (38% vs 16%) and 180 (25% vs 12%) compared with the SZC arm (Supplementary Fig. S3).

## DISCUSSION

In this randomized, controlled, open-label study comparing continuous SZC versus SOC after sK^+^ normalization in patients with CKD who were admitted to hospital with hyperkalaemia, no statistically significant difference was observed in the primary endpoint of normokalaemia responder at 180 days post-discharge.

The current results are unexpected given that previous studies have demonstrated the short-term efficacy of SZC in lowering sK^+^ levels over 4 weeks and maintaining normokalaemia in an outpatient setting for up to 12 months [16, 18, 24]. Consequently, all aspects of the study were scrutinized to elucidate possible reasons for the findings, and a series of post hoc, hypothesis-generating analyses were performed to further identify response trends that might add to patient care.

Key study design and logistical limitations were identified. These include the broad definition of normokalaemia non-response; the high rate of missing sK^+^ data at 180 days, due to a relatively narrow interval for the 180-day sK^+^ measurement that may not have been practical for real-world evaluation at all study centres; a baseline RAASi imbalance; and limited geographic generalizability, which may have masked the true treatment effects. These limitations, within the context of the post hoc analysis, are discussed in detail below.

The normokalaemia non-response criteria were very broad; all of the following counted as non-responses: a participant’s end-of-treatment sK^+^ reading (180 days post-discharge) outside of the 1-week visit window, use of rescue therapy, down-titration or discontinuation of RAASi, death, lost to follow-up, participants who withdrew, and site decision to withdraw participants. However, this may have led to the misclassification of participants who otherwise achieved normokalaemia as treatment failures, which hinders interpretation of the primary findings. In particular, participant withdrawal of consent, study site decision to withdraw participants, death, and participants lost to follow-up (Fig. 1B) may be inappropriate reasons for normokalaemia non-response, as they result from a range of events that may not otherwise have impacted the primary endpoint. The number of participants enrolled in the study provided adequate power for assessment of the primary study endpoint. However, high and imbalanced levels of missing sK^+^ data at 180 days led to overall levels of response that were substantially lower than the original assumptions in the sample size calculations used to power the study. Accordingly, the primary endpoint of normokalaemia response may not reflect the true biochemical efficacy of SZC observed previously in clinical trials, and the absence of statistically significant findings cannot be interpreted as evidence of no effect.

Over half of participants in the SZC group were without a valid laboratory sK^+^ reading for the primary analysis, most commonly because the reading was taken outside the 1-week visit window specified in the protocol (potentially owing to practical challenges). When those participants were considered, the results were numerically in favour of SZC, highlighting the impact of the excluded sK^+^ data on the study’s ability to detect meaningful differences in the response endpoint. As part of the post hoc sensitivity analyses, sK^+^ readings were analysed at 90 days, when there were fewer missing sK^+^ readings. While not statistically significant, the observed numerically higher proportion of responders in the SZC versus SOC arm at 90 days, particularly in the per-protocol set, is informative and may offer a more reliable estimate of treatment effect compared with the highly impacted 180-day endpoint, which suffered from both substantial and imbalanced missingness. However, the results from these analyses must be interpreted in the context of their post hoc nature and nominal *P* values.

There was a clear imbalance in ACEi/ARB and MRA prescribing between the randomized groups, with higher prescribing observed in the SZC arm at randomization and throughout the study versus the SOC arm. Additionally, twice the number of participants in the SZC arm were at target RAASi dose at randomization compared with the SOC arm. The exact extent to which the consistent imbalance in RAASi use confounded the primary findings is unknown. This imbalance suggests a potential selection and management bias on the part of the treating physician. The open-label nature of this study may have influenced the treating physicians’ prescribing decisions; both participants and healthcare providers were aware of the treatment allocations (SZC or SOC) following hospital discharge, and RAASi medications may have been prescribed after randomization. This is likely to have driven the higher RAASi prescribing rate in the SZC arm where the risk of hyperkalaemia would be lessened. Such prescribing trends align with previous studies that have demonstrated the ability of SZC to enable continuation and optimization of RAASi therapy by achieving and maintaining normokalaemia [18, 20, 21]. In the OPTIMIZE I real-world evidence study, almost 80% of participants who were treated with SZC for hyperkalaemia maintained or up-titrated their RAASi dose during follow-up [20]. In the ZORA real-world evidence study across the United States, Japan, and Spain, participants treated with SZC were substantially more likely to maintain guideline-concordant RAASi therapy following hyperkalaemia compared with participants not receiving a K^+^ binder [25]. By stabilizing sK^+^ levels, treatment with SZC may enable clinicians to adhere to international guideline recommendations to initiate or up-titrate RAASi therapy to maximally effective doses, thereby improving cardiorenal outcomes in the long-term [11]. At a population level, increasing the proportion of patients who can receive guideline-directed RAASi treatment for CKD will likely prevent a substantial number of cardiovascular events and deaths [7]. For patients who experience recurrent hyperkalaemia, there is a concern that RAASi therapy may never be fully optimized, ultimately compromising long-term cardiorenal outcomes [12–14].

Despite the higher RAASi use and dose in the SZC arm, time to first occurrence of a hyperkalaemia-related event trended sooner (although not significantly so) in the SOC arm, suggesting that SZC was contributing to the prevention of such events. This is supported by the lower proportion of participants in the SZC arm who experienced hyperkalaemia at days 90 and 180.

At randomization, there was a numerically higher proportion of participants with CKD stage 4/5 in the SZC arm than in the SOC arm. Whether this randomization bias influenced the proportion of participants who achieved normokalaemia in the SZC arm is unknown.

Finally, a very high proportion of the study population (85%) was recruited from Spain, and only 30% of randomized participants were female. There are limited data for post-discharge outcomes following hyperkalaemia [26, 27], and so differences may exist between country-specific healthcare systems and discharge practices, and by gender. These factors limit the generalizability of the findings when considered in the context of a wider geographical and female population.

There were numerically more deaths in the SZC arm than the SOC arm, although all deaths were adjudicated to be unrelated to the study medication. Differences in SAEs between the SZC and SOC arms were most prominent for gastrointestinal disorders (5.9% vs 1.5% of participants, respectively); of note, all gastrointestinal disorder SAEs occurred in *n* = 1 participants. Overall, these results for SZC are consistent with its established safety profile, with no new safety findings [17, 18].

In conclusion, the study did not meet its primary endpoint. However, numerous study design limitations were identified, which may have masked true treatment effects. The positive trends observed in our post hoc analyses suggest potential benefits of continued treatment with SZC in the outpatient setting in all-cause hospitalized patients with CKD and hyperkalaemia. Further, these findings underscore the complexity of long-term hyperkalaemia management following hospital discharge. Finally, this study highlights important lessons for future trial design concerning endpoint definition and handling of missing data, which could inadvertently lead to the omission of results that are clinically meaningful.

## Supplementary Material

sfag158_Supplemental_File

## Data Availability

Data underlying the findings described in this manuscript may be requested in accordance with AstraZeneca’s data sharing policy described at https://www.astrazenecaclinicaltrials.com/our-transparency-commitments/. AstraZeneca Group of Companies allows researchers to submit a request to access anonymized patient-level clinical data, aggregate clinical or genomics data (when available), and anonymized clinical study reports through the Vivli web-based data request platform.

## References

[1] Fried L, Kovesdy CP, Palmer BF. New options for the management of chronic hyperkalemia. Kidney Int Suppl (2011). 2017;7:164–70. 10.1016/j.kisu.2017.09.001 30675431 PMC6341013

[2] Valdivielso JM, Balafa O, Ekart R et al. Hyperkalemia in chronic kidney disease in the new era of kidney protection therapies. Drugs. 2021;81:1467–89. 10.1007/s40265-021-01555-5 34313978

[3] Seliger SL . Hyperkalemia in patients with chronic renal failure. Nephrol Dial Transplant. 2019;34:iii12–iii8. 10.1093/ndt/gfz231 31800076

[4] Logan Ellis H, Al-Agil M, Kelly PA et al. The burden of hyperkalaemia on hospital healthcare resources. Clin Exp Med. 2024;24:190. 10.1007/s10238-024-01452-7 39136879 PMC11322248

[5] Humphrey TJL, James G, Wittbrodt ET et al. Adverse clinical outcomes associated with RAAS inhibitor discontinuation: analysis of over 400 000 patients from the UK Clinical Practice Research Datalink (CPRD). Clin Kidney J. 2021;14:2203–12. 10.1093/ckj/sfab029 34804520 PMC8598122

[6] Floege J, Frankel AH, Erickson KF et al. The burden of hyperkalaemia in chronic kidney disease: a systematic literature review. Clin Kidney J. 2025;18:sfaf127. 10.1093/ckj/sfaf127 40385591 PMC12082095

[7] Xie X, Liu Y, Perkovic V et al. Renin-angiotensin system inhibitors and kidney and cardiovascular outcomes in patients with CKD: a Bayesian network meta-analysis of randomized clinical trials. Am J Kidney Dis. 2016;67:728–41. 10.1053/j.ajkd.2015.10.011 26597926

[8] Karaca C, Ozcan SG. Hyperkalemia and use of RAAS inhibitors in stage 3-5 chronic kidney disease non-dialysis patients: a single-center experience. Eurasian J Med Invest. 2023;7:378–83. 10.14744/ejmi.2023.50258

[9] Epstein M, Reaven NL, Funk SE et al. Evaluation of the treatment gap between clinical guidelines and the utilization of renin-angiotensin-aldosterone system inhibitors. Am J Manag Care. 2015;21:S212–S20. 26619183

[10] Epstein M . Hyperkalemia constitutes a constraint for implementing renin-angiotensin-aldosterone inhibition: the widening gap between mandated treatment guidelines and the real-world clinical arena. Kidney Int Suppl (2011). 2016;6:20–8. 10.1016/j.kisu.2016.01.004 30675416 PMC6340904

[11] Kidney Disease: Improving Global Outcomes (KDIGO) CKD Work Group . KDIGO 2024 clinical practice guideline for the evaluation and management of chronic kidney disease. Kidney Int. 2024;105:S117–314. 10.1016/j.kint.2023.10.018 38490803

[12] Kanda E, Rastogi A, Murohara T et al. Clinical impact of suboptimal RAASi therapy following an episode of hyperkalemia. BMC Nephrol. 2023;24:18. 10.1186/s12882-022-03054-5 36658531 PMC9854063

[13] Linde C, Bakhai A, Furuland H et al. Real-world associations of renin-angiotensin-aldosterone system inhibitor dose, hyperkalemia, and adverse clinical outcomes in a cohort of patients with new-onset chronic kidney disease or heart failure in the United Kingdom. J Am Heart Assoc. 2019;8:e012655. 10.1161/JAHA.119.012655 31711387 PMC6915283

[14] Svensson MK, Murohara T, Lesén E et al. Hyperkalaemia-related reduction of RAASi treatment associates with more subsequent inpatient care. Nephrol Dial Transplant. 2024;39:1258–67. 10.1093/ndt/gfae016 38253386 PMC11334062

[15] Stavros F, Yang A, Leon A et al. Characterization of structure and function of ZS-9, a K^+^ selective ion trap. PLoS One. 2014;9:e114686. 10.1371/journal.pone.0114686 25531770 PMC4273971

[16] Zannad F, Hsu BG, Maeda Y et al. Efficacy and safety of sodium zirconium cyclosilicate for hyperkalaemia: the randomized, placebo-controlled HARMONIZE-Global study. ESC Heart Fail. 2020;7:54–64. 31944628 10.1002/ehf2.12561 PMC7083449

[17] Roger SD, Lavin PT, Lerma EV et al. Long-term safety and efficacy of sodium zirconium cyclosilicate for hyperkalaemia in patients with mild/moderate versus severe/end-stage chronic kidney disease: comparative results from an open-label, phase 3 study. Nephrol Dial Transplant. 2021;36:137–50. 10.1093/ndt/gfz285 32030422 PMC7771984

[18] Spinowitz BS, Fishbane S, Pergola PE et al. Sodium zirconium cyclosilicate among individuals with hyperkalemia: a 12-month phase 3 study. Clin J Am Soc Nephrol. 2019;14:798–809. 10.2215/CJN.12651018 31110051 PMC6556727

[19] Gnesi M, Daniel F, Mongelli V et al. The role of sodium zirconium cyclosilicate drug utilization in managing hyperkalemia: impact on healthcare resource utilization and on maintenance of renin-angiotensin-aldosterone system inhibitor therapy in Italian clinical practice. J Med Econ. 2025;28:576–85. 10.1080/13696998.2025.2487357 40244700

[20] Agiro A, An A, Cook EE et al. Real-world modifications of renin-angiotensin-aldosterone system inhibitors in patients with hyperkalemia initiating sodium zirconium cyclosilicate therapy: the OPTIMIZE I study. Adv Ther. 2023;40:2886–901. 10.1007/s12325-023-02518-w 37140706 PMC10220114

[21] Agiro A, Greatsinger A, Mu F et al. Renin-angiotensin-aldosterone system inhibitor dosing after initiation of outpatient sodium zirconium cyclosilicate therapy: the GALVANIZE RAASi real-world evidence study. Adv Ther. 2025;42:3960–77. 10.1007/s12325-025-03254-z 40531443 PMC12313742

[22] ClinicalTrials.gov. NCT05347693, Continuing Sodium Zirconium Cyclosilicate (SZC) After Discharge Study (CONTINUITY). https://clinicaltrials.gov/study/NCT05347693 (Date accessed 23 March 2026).

[23] Burton JO, Allum AM, Amin A et al. Rationale and design of CONTINUITY: a Phase 4 randomized controlled trial of continued post-discharge sodium zirconium cyclosilicate treatment versus standard of care for hyperkalemia in chronic kidney disease. Clin Kidney J. 2023;16:1160–9. 10.1093/ckj/sfad053 37398685 PMC10310508

[24] Liang X, Lu W, Yu X et al. HARMONIZE Asia: a Phase III randomized study to investigate the efficacy and safety of sodium zirconium cyclosilicate in patients with hyperkalemia in China. Clin Ther. 2024;46:702–10. 10.1016/j.clinthera.2024.07.004 39112102

[25] Rastogi A, Pollack CVJ, Sánchez Lázaro IJ et al. Maintained renin–angiotensin–aldosterone system inhibitor therapy with sodium zirconium cyclosilicate following a hyperkalaemia episode: a multi-country cohort study. Clin Kidney J. 2024;17:sfae083. 10.1093/ckj/sfae083 38699484 PMC11062025

[26] Davis J, Israni R, Mu F et al. Inpatient management and post-discharge outcomes of hyperkalemia. Hosp Pract (1995). 2021;49:273–9. 10.1080/21548331.2021.1925554 34038312 PMC9102837

[27] Betts KA, Woolley JM, Mu F et al. Postdischarge health care costs and readmission in patients with hyperkalemia-related hospitalizations. Kidney Int Rep. 2020;5:1280–90. 10.1016/j.ekir.2020.06.004 32775827 PMC7403556

