# Supplementary material for: Sodium zirconium cyclosilicate post hospital discharge to prevent hyperkalaemia: phase 4, randomized CONTINUITY trial
Source: Clin Kidney J. 2026 May 19;19(6):sfag158. doi: 10.1093/ckj/sfag158 (PMC13284702; doi:10.1093/ckj/sfag158)
Supplement: sfag158_Supplemental_File [file sfag158_supplemental_file.docx]

**Sodium zirconium cyclosilicate post hospital discharge to prevent hyperkalaemia: phase 4, randomized CONTINUITY trial**

**James O. Burton^1^, María J. Izquierdo^2^, Cecilia Linde^3^, Nicolás R. Robles^4^, Manish M. Sood^5^, Alaster M. Allum^6^, James M. Eudicone^7^, Magnus Dahl^8^, Alpesh N. Amin^9^**

*^1^Division of Cardiovascular Sciences, University of Leicester and University Hospitals of Leicester, Leicester, UK; ^2^Head of Nephrology Department, University Hospital of Burgos, Burgos, Spain; ^3^Department of Cardiology, Karolinska Institutet, Stockholm, Sweden; ^4^Universidad de Extremadura, Head Nephrology Department and Hypertension Unit, Hospital Universitario de Badajoz, Badajoz, Spain; ^5^Ottawa Hospital Research Institute, The Ottawa Hospital, Ottawa, Canada; ^6^BioPharmaceuticals Medical, AstraZeneca, Cambridge, UK; ^7^BioPharmaceuticals Medical Evidence, AstraZeneca, Wilmington, DE, USA; ^8^BioPharmaceuticals Medical, AstraZeneca, Gothenburg, Sweden; ^9^Department of Medicine, University of California Irvine, Irvine, CA, USA*

**Correspondence to:** James O. Burton; E-mail: jb343@leicester.ac.uk

# Table of Contents

[Supplementary Methods 3](#_Toc225174218)

[Table S1: Comorbidities at enrolment (occurring in at least 5% of participants in either treatment group) (safety set randomized). 5](#_Toc225174219)

[Table S2: Proportion of patients on target/some/no dose of ACEi/ARB and MRA at randomization, 90 days and 180 days 7](#_Toc225174220)

[Figure S1: Key updates to study design 8](#_Toc225174221)

[Figure S2: Participant disposition (screened set) 9](#_Toc225174222)

[Figure S3: Proportion of participants with hyperkalaemia (full analysis set) 10](#_Toc225174223)

# Supplementary Methods

To allow for the inclusion of a wider and more applicable patient population, the following key protocol updates were made: allowance for enrolment of patients with a chronic kidney disease (CKD) diagnosis (any stage) or estimated glomerular filtration rate (eGFR) <90 mL/min/1.73 m^2^ at, or within 3 months of, study screening (**Fig. S1**). Previously, the study included only patients with stage 3b to 5 CKD and/or with eGFR <45 mL/min/1.73 m^2^. While hyperkalaemia is less common in patients with early-stage CKD, these patients may have already experienced a hyperkalaemia episode, may be receiving renin-angiotensin-aldosterone system inhibitor (RAASi) therapy, and may be at high risk of hyperkalaemia recurrence. Additionally, the inclusion criteria were clarified such that patients who were treated for a current hyperkalaemia episode, as defined by the study site’s local practice and serum potassium (sK^+^) ≤6.5 mmol/L, could be normokalaemic (sK^+^ 3.5–5.0 mmol/L) at the time of enrolment.

As part of the protocol amendments, patients were excluded if they were receiving ongoing treatment with sodium zirconium cyclosilicate (SZC) or patiromer before the current emergency department (ED) visit/hospitalization (rather than excluding patients who were receiving any K^+^ binder treatment before the current ED visit/hospitalization, per the original protocol). Furthermore, the protocol was updated to allow initiation of SZC or patiromer during the current ED visit/hospitalization, while previously, initiation of any K^+^ binder therapy had not been allowed. Emergency/unscheduled haemodialysis to treat hyperkalaemia during the current hospitalization was permitted as part of the protocol updates.

In the protocol update, the definition of the first secondary endpoint, ‘time to first occurrence of hospital admission or ED visit with hyperkalaemia as a contributing factor’ was updated to ‘time to first occurrence of all-cause hospital admission or ED visit with hyperkalaemia as a contributing factor’ (**Fig. S1**).

Table S1: Comorbidities at enrolment (occurring in at least 5% of participants in either treatment group) (safety set randomized)

| System organ class/ preferred term | *N* (%) | | |
| --- | --- | --- | --- |
|  | SZC (*n* = 68) | SOC (*n* = 68) | Total (*N* = 136) |
| Infections and infestations | 12 (17.6) | 13 (19.1) | 25 (18.4) |
| Neoplasms benign, malignant and unspecified (including cysts and polyps) | 20 (29.4) | 21 (30.9) | 41 (30.1) |
| Blood and lymphatic system disorders | 19 (27.9) | 24 (35.5) | 43 (31.6) |
| Anaemia | 12 (17.6) | 13 (19.1) | 25 (18.4) |
| Endocrine disorders | 16 (23.5) | 12 (17.6) | 28 (20.6) |
| Hypothyroidism | 6 (8.8) | 6 (8.8) | 12 (8.8) |
| Metabolism and nutrition disorders | 65 (95.6) | 60 (88.2) | 125 (91.9) |
| Diabetes  (mellitus/type 1/type 2/steroid) | 44 (64.7) | 39 (57.4) | 83 (60.6) |
| Dyslipidaemia | 40 (58.8) | 31 (45.6) | 71 (52.2) |
| Hypercholesterolaemia | 6 (8.8) | 6 (8.8) | 12 (8.8) |
| Hyperuricaemia | 18 (26.5) | 21 (30.9) | 39 (28.7) |
| Metabolic acidosis | 4 (5.9) | 3 (4.4) | 7 (5.1) |
| Obesity | 8 (11.8) | 4 (5.9) | 12 (8.8) |
| Vitamin D deficiency | 11 (16.2) | 9 (13.2) | 20 (14.7) |
| Psychiatric disorders | 19 (27.9) | 18 (26.5) | 37 (27.2) |
| Depression | 6 (8.8) | 8 (11.8) | 14 (10.3) |
| Insomnia | 5 (7.4) | 4 (5.9) | 9 (6.6) |
| Neurological disorders | 21 (30.9) | 16 (23.5) | 37 (27.2) |
| Cardiac disorders | 37 (54.4) | 43 (63.2) | 80 (58.8) |
| Atrial fibrillation | 17 (25.0) | 21 (30.9) | 38 (27.9) |
| Heart failure | 15 (22.1) | 14 (20.6) | 29 (21.3) |
| Myocardial ischaemia | 13 (19.1) | 11 (16.2) | 24 (17.6) |
| Vascular disorders | 63 (92.6) | 62 (91.2) | 125 (91.9) |
| Hypertension | 62 (91.2) | 60 (88.2) | 122 (89.7) |
| Respiratory, thoracic, and mediastinal disorders | 28 (41.2) | 26 (38.2) | 54 (39.7) |
| COPD | 13 (19.1) | 14 (20.6) | 27 (19.9) |
| Obstructive sleep apnoea syndrome | 8 (11.8) | 1 (1.5) | 9 (6.6) |
| Gastrointestinal disorders | 20 (29.4) | 17 (25.0) | 37 (27.2) |
| Renal and urinary disorders | 66 (97.1) | 67 (98.5) | 133 (97.8) |
| CKD | 62 (91.2) | 64 (94.1) | 126 (92.6) |

CKD, chronic kidney disease; COPD, chronic obstructive pulmonary disease; SOC, standard of care; SZC, sodium zirconium cyclosilicate.

Table S2: Proportion of patients on target/some/no dose of ACEi/ARB and MRA at randomization, 90 days and 180 days

| *n* (%) | SZC (*n* = 68) | SOC (*n* = 68) | Total (*N* = 136) |
| --- | --- | --- | --- |
| At randomization |  |  |  |
| Target dose | 14 (20.6) | 6 (8.8) | 20 (14.7) |
| Some dose | 37 (54.4) | 41 (60.3) | 78 (57.4) |
| No dose | 17 (25.0) | 21 (30.9) | 38 (27.9) |
| At 90 days |  |  |  |
| Target dose | 12 (17.6) | 7 (10.3) | 19 (14.0) |
| Some dose | 40 (58.8) | 41 (60.3) | 81 (59.6) |
| No dose | 16 (23.5) | 20 (29.4) | 36 (26.5) |
| At 180 days |  |  |  |
| Target dose | 13 (19.1) | 6 (8.8) | 19 (14.0) |
| Some dose | 40 (58.8) | 40 (58.8) | 80 (58.8) |
| No dose | 15 (22.1) | 22 (32.4) | 37 (27.2) |

ACEi, angiotensin-converting enzyme inhibitor; ARB, angiotensin receptor blocker; MRA, mineralocorticoid receptor antagonist; SOC, standard of care; SZC, sodium zirconium cyclosilicate.

Figure S1: Key updates to study design


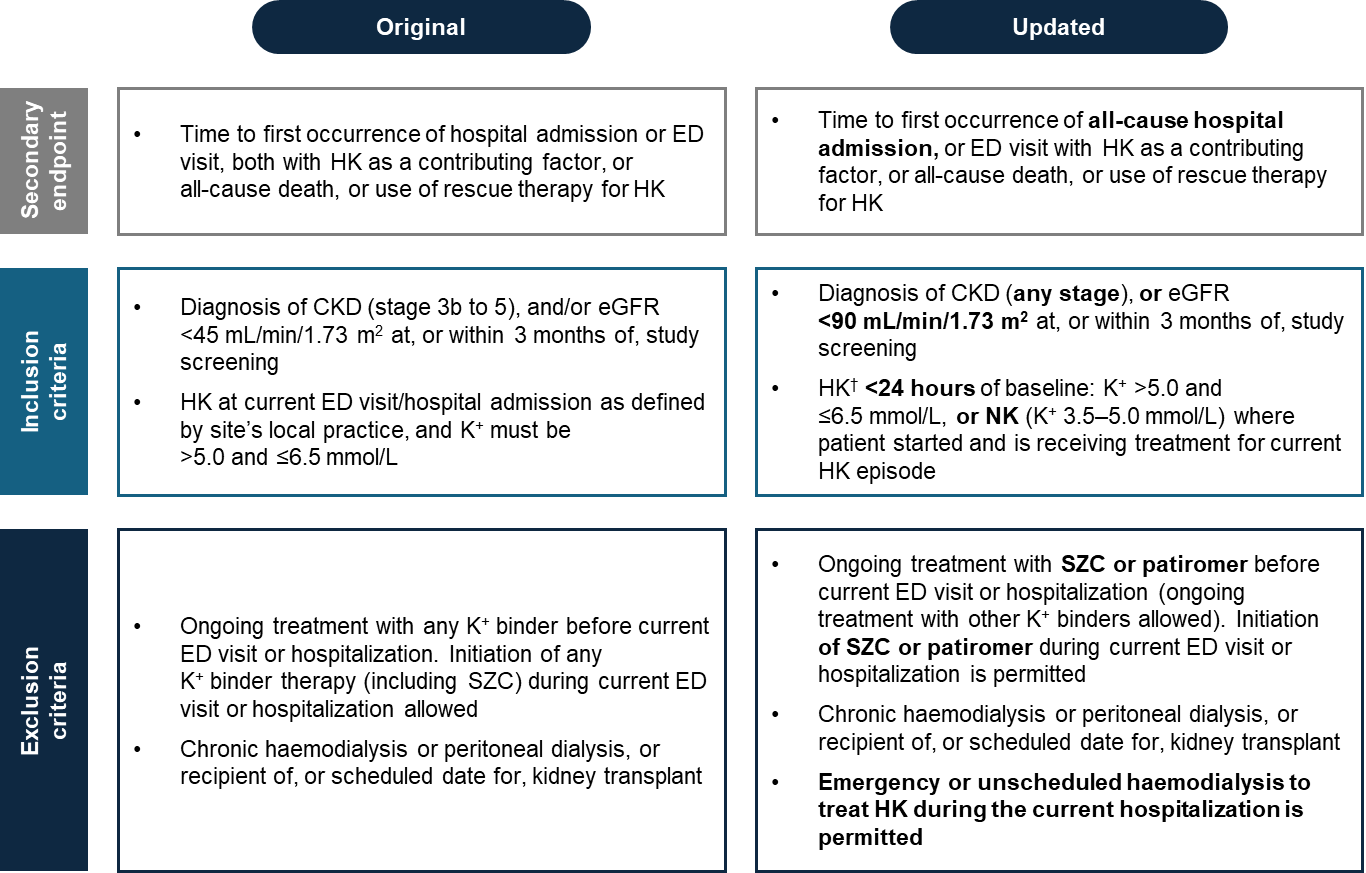


^†^Local laboratory K^+^ measurement.

CKD, chronic kidney disease; ED, emergency department; eGFR, estimated glomerular filtration rate; HK, hyperkalaemia; NK, normokalaemia; SZC, sodium zirconium cyclosilicate.

Figure S2: Participant disposition (screened set)


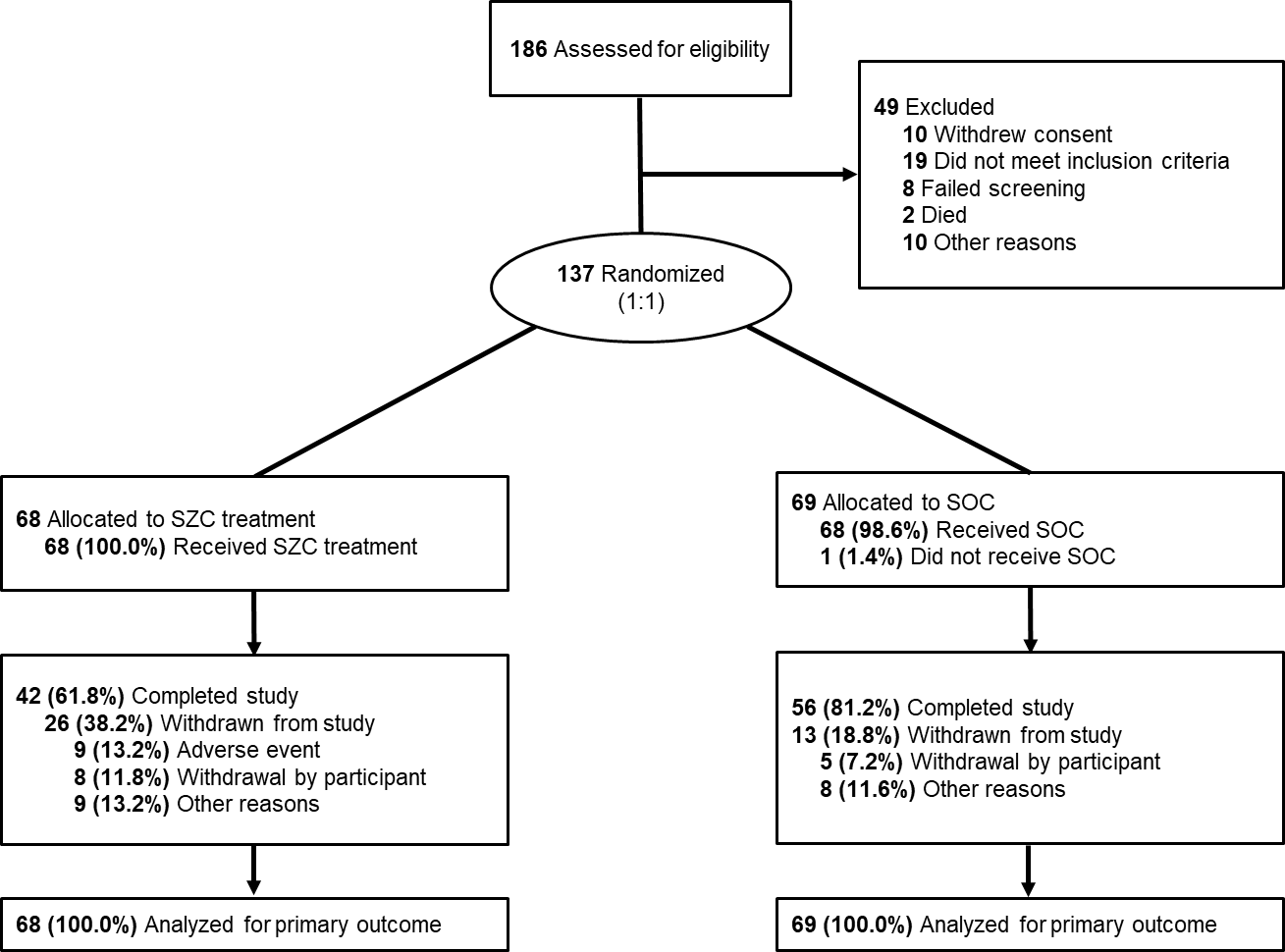


SOC, standard of care; SZC, sodium zirconium cyclosilicate.

Figure S3: Proportion of participants with hyperkalaemia (full analysis set)


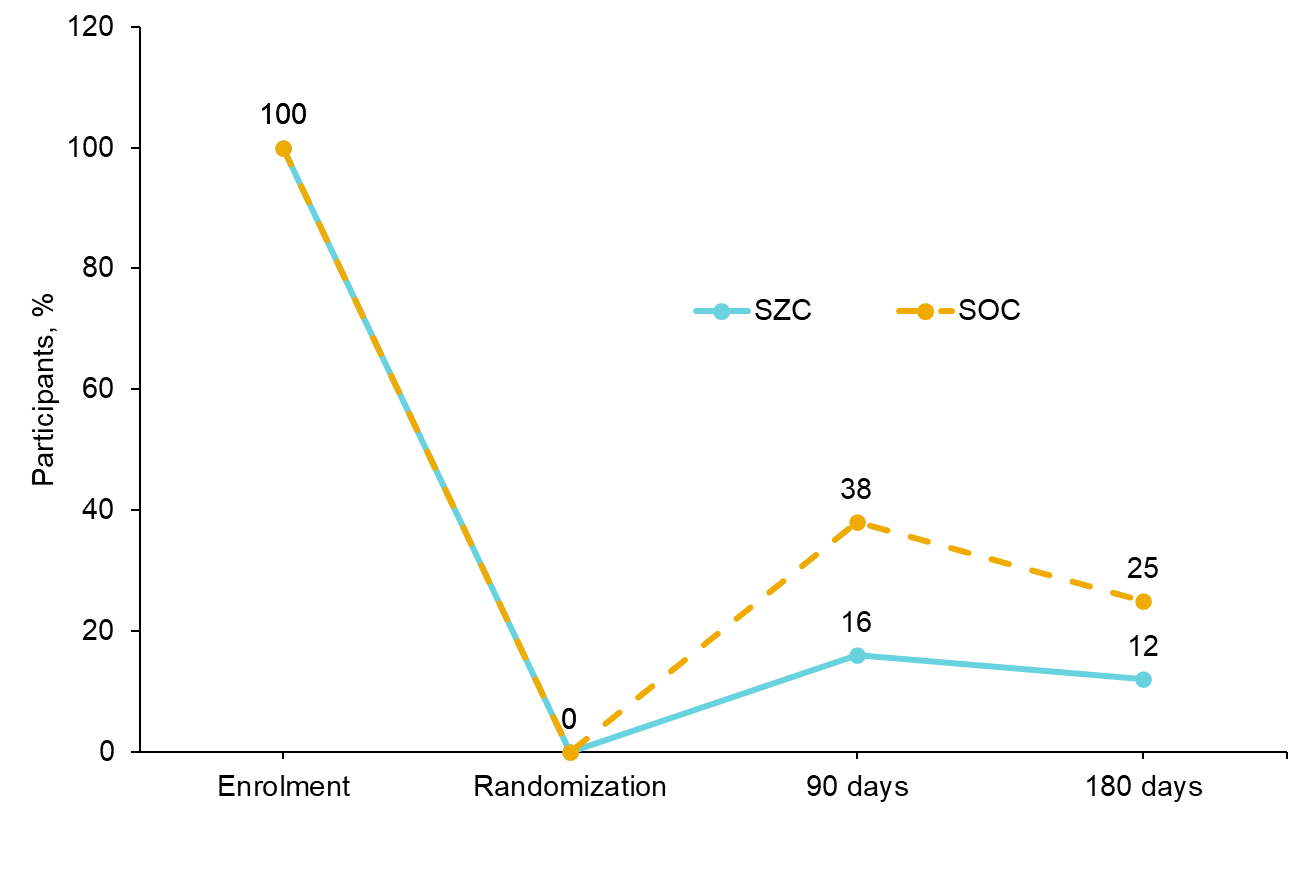


SOC, standard of care; SZC, sodium zirconium cyclosilicate.
